# Assessment of hepatocellular carcinoma risk based on peg-interferon plus ribavirin treatment experience in this new era of highly effective oral antiviral drugs: Erratum

**DOI:** 10.1097/MD.0000000000006958

**Published:** 2017-05-19

In the article, “Assessment of hepatocellular carcinoma risk based on peg-interferon plus ribavirin treatment experience in this new era of highly effective oral antiviral drugs”,^[1]^ which appeared in Volume 96, Issue 1 of *Medicine*, the information about the study funding appeared incorrectly as “This study was supported by an Inha University Hospital grant.” It should have appeared as “This study was supported by an Inha University Research grant.”
